# Orbital metastases from prostate adenocarcinoma: Case report and review of the literature

**DOI:** 10.1016/j.amsu.2022.103530

**Published:** 2022-03-29

**Authors:** O. Nabih, N. Mtalai, L. EL Maaloum, B. Allali, A. EL kettani

**Affiliations:** a Medical Resident at Pediatric Ophthalmology Department, Hopital 20 Aout, 1953, Casablanca, Morocco; b Pediatric Ophthalmology Department, Hopital 20 Aout, 1953, Casablanca, Morocco

**Keywords:** Orbital metastases, Prostate cancer, Malignant tumor, Hormonal therapy

## Abstract

**Introduction:**

Prostate carcinoma metastasizes usually to lymph nodes and bone. Its metastases to the orbital cavity remain very rare.

**Observation:**

We report here the case of an 80-year-old man diagnosed with a non-metastatic prostate adenocarcinoma 9 months earlier, who was found to have an orbital metastasis revealed by a proptosis of his left eye. He received hormonal therapy, chemotherapy and radiotherapy.

**Discussion:**

Orbital metastases from prostate carcinoma have many similarities to other orbital metastases in their presentation. Their diagnosis is easily done when there is a history of a primary tumor. Presenting symptoms include proptosis, limitation of eye movements, diplopia and decreased vision.

**Conclusion:**

Through this case report and a review of literature, we discuss the incidence, the clinical presentation and the management of these tumors.

## 1 Introduction

Orbital metastases from prostate adenocarcinoma are very rare. In some cases, these metastases can be the initial presenting sign of prostate cancer [1]. Therefore, the role of the ophthalmologist in the diagnosis of these tumors is very important.

We report the case of an 80-year-old man with history of prostate cancer who was diagnosed with orbital metastases.

## 2 Observation

We report the case of an 80-year-old man with a history of a located prostate adenocarcinoma, who went through a radical prostatectomy 9 months earlier with total PSA level was 500 ng/ml before surgery and prostate biopsy revealed poorly differentiated prostatic adenocarcinoma with a Gleason score of 9 (5 + 4).

During the last 3 months, an isolated unilateral exophthalmos OS was noticed by his spouse.

The clinical examination reported a patient in good general condition, with 8/10 P3 visual acuity in both eyes. The confrontation visual field testing was normal. The local examination revealed an axial non-pulsatile proptosis of the left eye.

The anterior segment and the fundus examination were normal in both eyes. Optic discs presented a large physiological cups.

**Fig. 1:**
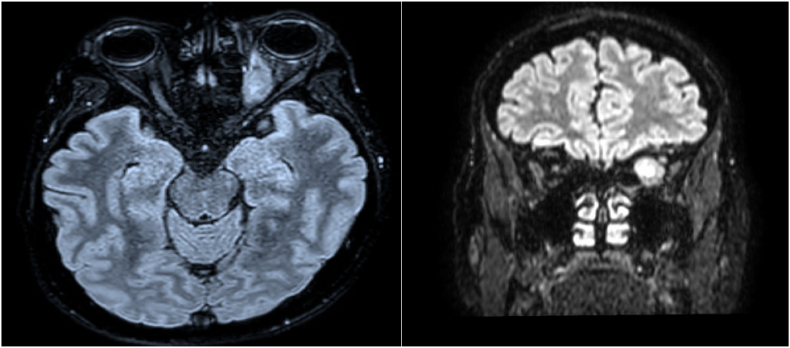
Axial and coronal flair sequences showing a left compressive mass that occupies the orbital cavity, well limited with regular contours that partially fills the intra-conical fat and compresses the optic nerve that is repressed laterally.

Cranial magnetic resonance imaging revealed a left compressive mass that occupies the orbital cavity, with a subperiosteal aspect, well limited with regular contours that partially fills the intra-conical fat and compresses the optic nerve and the inferior rectus muscle. It measures 32 × 24 × 49 mm. Fig. 1.

IRM are lead to the diagnosis by showing very suggestive osteoblastic lesions. Osteoclastic lesions are rarer, indicating an aggressive form at the terminal stage.

Spine radiology showed no vertebral bone demineralization.

The bone scintigraphy came back normal.

**Fig. 2:**
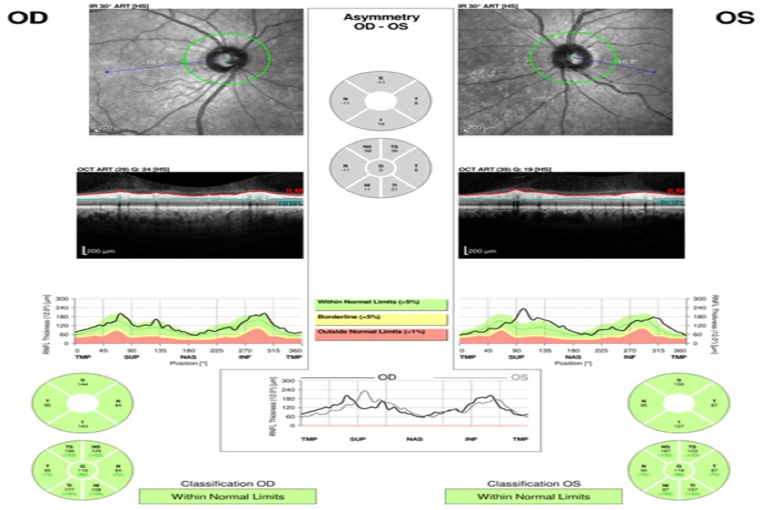
The papillary OCT did not reveal any loss of the nerve fiber layer.

The papillary optical coherence tomography was normal. Fig. 2.

The diagnosis of certainty is confirmed by cytological aspiration puncture of the orbital lesion with a fine needle with immunohistochemical study and immunostaining of PSA with peroxidase.

After a multidisciplinary team meeting and the approval of the patient, it was decided to start with a hormonal therapy-complete androgen blockade - associated with chemotherapy as well as radiotherapy. The patient developed brain metastases and died 8 months later.

## 3 Discussion

Prostate cancer, when metastatic, typically involves the axial skeleton and lymph nodes. Orbital metastases of prostate cancer are very rare [[2], [3], [4]].

In general, orbital metastases represent 6% of all the orbital tumors [5]. The predominant primary sites of the intraocular metastases are breast and lung [6,7].

Although metastases from prostate carcinoma to the ocular area have been considered extremely rare, prostate cancer was found to be the second most common primary site of orbital metastases in men [8,9]. These statistics might be underrated since that the orbital and intraocular metastases often remain subclinical [10].

During metastasis, tumor cells were found to get detached from the primary tumor and may intravasate into and disseminate through the blood circulation or lymphatic system [11].

Orbital metastases are less frequent than the ocular ones [12,13]. They are rarely bilateral [[8], [9], [10], [11], [12], [13], [14]]. Most of the time, orbital metastases are located exclusively in extraconal spaces (50%) and the rest are found either in intraconal spaces (30%) or in both (20%) [13].

Their localization in the orbit is variable, they seem to occupy the lateral (39%) and superior (32%) quadrants more frequently that the medial (20%) or the inferior (12%) ones.

In our case, the localization of the metastasis was the lateral quadrant, as in breast and lung most frequent metastasis’ location.

Studies showed that some primary tumors have preferences for certain tissues. Prostate carcinoma is more likely to metastasize to bone, whereas breast carcinoma tends to metastasize in orbital fat and muscle [13,14].

The first and most frequent clinical signs associated with orbital and ocular metastasis are the proptosis and the limitation of eye movements.

Studies showed that the most reported symptoms associated with orbital metastases are the diplopia, pain and decreased vision; whereas proptosis, motility disturbances and disk oedema are the most presenting signs [14].

The diagnosis of orbital metastases is easily done when there is a history of a primary tumor. In our case, the patient has already had a radical prostatectomy for a prostate adenocarcinoma and presented no other signs of another tumor. Biopsy should be considered in patients with less straightforward presentation in order to exclude second primary tumor.

When one suspects the presence of metastasis, fine-needle puncture and aspiration biopsy is indicated. It can provide anatomopathological diagnosis which can help locate the primary tumor. Its main complication is the possibility of diffusing tumoral cells. In our case, since there was a history of an adenocarcinoma, there was no need to do a cyto-biopsy of the tumor.

In general, orbital metastases are not an indication for surgical intervention since it is not a radical treatment [12,14].

If the orbital tumor gets symptomatic or increase in size, some treatments can be initiated such as radiotherapy, chemotherapy, or hormonal therapy for hormone-sensitive tumors such as breast and prostate cancers.

The prognosis of patients presenting orbital metastases is in general reserved and it depends on the type and location of the prior tumor. Out the 245 cases reported in medical literature, the mean rate survival since the detection of the metastasis was 9.3 months.

## 4 Conclusion

Orbital metastases of prostate tumors present one of the rarest metastatic sites. The role of the ophthalmologist in its early diagnosis is important to the general prognosis improvement, thus it remains reserved.

## Ethical approval

This type of study does not require any ethical approval by our institution.

## Sources of funding

This study did not receive any sources of funding.

## Author contribution

O.Nabih: drafting the article, study concept, writing the article. N.Mtalai: acquisition of data. L.El maaloum: study design. B.Allali: revising the article. A. El kettani: final approval.

## Registration of research studies

1. Name of the registry:

2. Unique identifying number or registration ID:

3. Hyperlink to your specific registration (must be publicly accessible and will be checked):

## Guarantor

O.Nabih.

## Consent

Patient provided written, retrospective consent for publication following detailed explanation of the purpose of manuscript and understanding that no identifiable information was going to be released.

## Funding

This research did not receive any specific grant(s) from funding agencies in the public, commercial, or not-for-profit sectors.

## Provenance and peer review

Not commissioned, externally peer-reviewed.

## Declaration of competing interest

The authors declare no conflict of interest.
